# A free weekly iron-folic acid supplementation and regular deworming program is associated with improved hemoglobin and iron status indicators in Vietnamese women

**DOI:** 10.1186/1471-2458-9-261

**Published:** 2009-07-24

**Authors:** Gerard J Casey, Tran Q Phuc, Lachlan MacGregor, Antonio Montresor, Seema Mihrshahi, Tran D Thach, Nong T Tien, Beverley-Ann Biggs

**Affiliations:** 1 Department of Medicine (RMH/WH), The University of Melbourne, The Royal Melbourne Hospital, Parkville 3050, Australia; 2 Department of Parasitology, National Institute of Malariology, Parasitology and Entomology (NIMPE), Hanoi, Vietnam; 3 Clinical Epidemiology & Health Service Evaluation Unit, Royal Melbourne Hospital, Parkville 3050, Australia; 4 Public Health Specialist, World Health Organization, Hanoi, Vietnam; 5 Research And Training Centre for Community Development, 39 Lane 255, Vong St., Hanoi, Vietnam; 6 6 Department of Malaria Treatment and Research, National Institute of Malariology, Parasitology and Entomology (NIMPE), Hanoi, Vietnam; 7 Centre of Clinical Research Excellence in Infectious Diseases (CCREID), The Royal Melbourne Hospital, Parkville 3050, Australia

## Abstract

**Background:**

Anemia due to iron deficiency is recognized as one of the major nutritional deficiencies in women and children in developing countries. Daily iron supplementation for pregnant women is recommended in many countries although there are few reports of these programs working efficiently or effectively. Weekly iron-folic acid supplementation (WIFS) and regular deworming treatment is recommended for non-pregnant women living in areas with high rates of anemia. Following a baseline survey to assess the prevalence of anemia, iron deficiency and soil transmitted helminth infections, we implemented a program to make WIFS and regular deworming treatment freely and universally available for all women of reproductive age in two districts of a province in northern Vietnam over a 12 month period. The impact of the program at the population level was assessed in terms of: i) change in mean hemoglobin and iron status indicators, and ii) change in the prevalence of anemia, iron deficiency and hookworm infections.

**Method:**

Distribution of WIFS and deworming were integrated with routine health services and made available to 52,000 women. Demographic data and blood and stool samples were collected in baseline, and three and 12-month post-implementation surveys using a population-based, stratified multi-stage cluster sampling design.

**Results:**

The mean Hb increased by 9.6 g/L (95% CI, 5.7, 13.5, p < 0.001) during the study period. Anemia (Hb<120 g/L) was present in 131/349 (37.5%, 95% CI 31.3, 44.8) subjects at baseline, and in 70/363 (19.3%, 95% CI 14.0, 24.6) after twelve months. Iron deficiency reduced from 75/329 (22.8%, 95% CI 16.9, 28.6) to 33/353 (9.3%, 95% CI 5.7, 13.0) by the 12-mnth survey, and hookworm infection from 279/366 (76.2%,, 95% CI 68.6, 83.8) to 66/287 (23.0%, 95% CI 17.5, 28.5) over the same period.

**Conclusion:**

A free, universal WIFS program with regular deworming was associated with reduced prevalence and severity of anemia, iron deficiency and hookworm infection when made available to Vietnamese women over a 12-month period.

## Background

Anemia affects more than 1.5 billion people world-wide. Women are at particular risk, with 45.7% of non-pregnant women of reproductive age (WRA) in South-East Asia and 47.5% in Africa reported as anemic [1]. Anemia and the primary cause, iron deficiency, lead to reduced physical capacity, with consequences for both social and economic development [2]. Women who become pregnant while anemic due to insufficient iron stores are at higher risk of preterm delivery [3]. Maternal anemia is also linked with an increased risk of maternal death, impaired fetal growth, low birth weight and increased neonatal mortality [4-7]. Maternal iron deficiency anemia has been shown to be a predictor of iron deficiency in infants [8], which in turn is a risk factor for adverse physical and cognitive development in childhood [9,10] and has implications for both maternal and infant mental health [11,12]. Low intake and poor absorption of dietary iron, and blood loss from menstruation and concurrent parasitic infections are major contributors to anemia and iron deficiency in women in less developed countries [7].

Food-based and supplementation strategies to improve iron status are recommended for populations at risk of anemia and iron deficiency [7]. Preventative weekly iron-folic acid supplementation (WIFS) for WRA is effective in improving iron stores [13-17] and has been advocated for widespread use in non-pregnant women in areas with high rates of maternal anemia [7]. This approach is supported by evidence that women with robust iron stores at the time of conception are less likely to develop iron deficiency anemia during pregnancy [18]. The current challenge is to develop implementation strategies and programmatic experience that can sustain universal long-term WIFS programs in resource-poor settings [19]. Vietnam initiated a daily iron supplementation program for pregnant women in 1993 [20]. Despite this, a survey undertaken in the central highlands of Vietnam in 2001 found that 62% of pregnant and 54% of non-pregnant women were anemic [21] following a trend in many countries that recommend similar regimens. We have previously reported 37.5% prevalence of anemia in non-pregnant women in northern Vietnam [22].

Following a baseline survey in November 2005 [22] we initiated a program that integrated distribution of universal WIFS and deworming into the existing health service structure in two districts of the northern mountainous province of Yen Bai, Vietnam, between May 2006 and April 2007. The program has subsequently been expanded to all districts of Yen Bai province. The broad aims were to identify the burden of anemia, iron deficiency, and soil transmitted helminth infections in non-pregnant WRA (reported in Pasricha et al 2008), and to investigate the impact of the WIFS/deworming intervention on hemoglobin (Hb), iron status indicators and hookworm prevalence. In this paper we report the impact of 12 months implementation of the WIFS/deworming intervention and report the results of a compliance monitoring survey conducted after three months of the intervention.

## Methods

### Study location and population

Yen Bai province, one of 64 provinces and cities in Vietnam, is a mountainous region with a largely rural economy, widespread poverty and diverse ethnic groups. Two districts, Yen Binh and Tran Yen, were chosen for the study. At the time each district had a total population of approximately 26,000 WRA. Women of reproductive age were defined in this setting as aged between 15 and 45 years and all were eligible for the intervention.

### Intervention

Before the intervention, two staff of each district Department of Preventive Medicine, two nurses from each commune health station and all village health workers in the two districts (a total of 680 health personnel) were trained about the causes, health risks, treatment and prevention of anemia and hookworm infection and received promotional and educational materials for the women. Commencing May 2006, weekly supplementation and 4 monthly deworming was implemented by active distribution through the existing health structure. Iron/folic acid and albendazole tablets were distributed from the provincial implementing agency, the Yen Bai Malaria Control Program office, through the district Preventive Medicine centres, to the Commune Health Stations (for albendazole treatment) and on to the Village Health Workers for distribution of the iron/folic acid tablets to the women on a monthly basis. In addition, health workers distributed simple educational materials. All WRA were encouraged to collect packs of four ferrous sulphate/folic acid tablets (60 mg/0.4 mg, UNICEF, Copenhagen) from their village health worker each month. Albendazole (400 mg, UNICEF, Copenhagen) was administered as observed treatment on locally designated days either at the commune health station or supervised in the village by a commune health worker.

### Sampling

A stratified multi-stage cluster sampling design was used for the baseline survey, which was conducted in November 2005. Primary sampling units (villages) were chosen using a 'probability proportional to size' random sampling method separately within each district, with half the target sample of villages taken from each district. Secondary sampling units (individual women) were selected randomly from each village using provincial lists. Sample size was calculated on an expected hemoglobin range of 90 g/L to 140 g/L with a population standard deviation of 10 g/L. Allowing for the clustered nature of the surveys a sample size of 250 was estimated to be sufficient to detect a 3 g/L change in the mean hemoglobin. Allowing for refusals to participate and potentially inaccessible villages, 34 villages were selected and 12 women from each village randomly selected, a total of 408 women.

Post-implementation surveys were conducted after three and 12 months of intervention using the same sampling design for choosing participants. We used a cross sectional survey approach for participant selection at each time point in order to examine the population effect of the intervention in the target districts.

### Samples and testing

All surveys were conducted by the same teams and included trained phlebotomists, stool preparation and analysis technicians, a demographic recorder and a supervisor. Sample collection and laboratory analysis has been previously reported [22] and was the same in each of the surveys. Briefly, Hb was assessed at the field site using a HemoCue 201+ (HemoCue AB, Angelholm, Sweden). A 3 mL sample of venous blood was also collected using a closed collection system into tubes containing fast clotting agent. Serum ferritin was measured using a sandwich immunoenzymatic assay (IEA; Beckman Coulter Access Reagents, Fullerton, CA). Soluble transferrin receptor was evaluated using enzyme-linked immunoassay (ELISA; IT; Orion Diagnostica, Espoo, Finland). The ratio of transferrin receptor to log (base 10) serum ferritin (TfR-F index) was calculated from these results. Anemia was defined as a hemoglobin concentration of <120 g/L and iron deficiency as serum ferritin of < 15 μg/L, except where otherwise stated, in concordance with WHO recommendations for women of reproductive age [7]. Transferrin receptor levels of 2.3 mg/L or above were considered abnormal, based on the manufacturer's reference interval (0.8–2.3 mg/L). The TfR-F index implies depletion of iron stores once the ratio exceeds 1.8 [23].

Faecal samples were examined microscopically for soil transmitted helminth eggs at the field site using standard Kato-Katz methodology [24]. Classification of hookworm infection was based on WHO guidelines of 0 eggs per gram (epg) as no infection, >0 and <2000 epg as mild infection and >2000 as moderate or severe infection. Soil transmitted helminth (STH) infection was categorized as positive if eggs of hookworm (*Ancylostoma duodenale *and *Necator americanus*), *Ascaris lumbricoides *or *Trichuris trichiura *were detected in the stool sample.

### Compliance monitoring

In July 2006, just prior to the 3-month post-implementation survey, the Research and Training Centre for Community Development (RTCCD), an independent Vietnamese non-Government Organization, monitored compliance in the two districts. Sampling was as described for the surveys above. Monitoring was conducted as one-to-one interviews with women and included questions about their understanding of anemia, iron deficiency and helminthiasis, the availability of the intervention and their recall of accessing the deworming treatment and taking the weekly iron tablets during the previous 10 weeks.

### Data entry and checking

Hb and helminth egg counts were entered into an Excel (Microsoft Office 2003) spreadsheet at the field site. Team supervisors crosschecked entries each day.

### Statistical analysis

The study sample was defined as randomly selected groups at baseline and three and twelve months post-implementation. Hb values were approximately normally distributed. Serum ferritin and soluble transferrin receptor values were right-skewed and so log-transformed for analysis. For certain multi-level ordered categorical variables (e.g. education level, place of work), some categories were collapsed to identify relevant differences. Robust confidence intervals were derived by clustering for village, the primary sampling unit.

Linear regression analysis was used for continuous variables while binary variables were analysed by logistic regression. For continuous variables the differences in means between time points were calculated in absolute values for Hb, and for the log transformed variables (serum ferritin, soluble transferrin receptor and the TfR/F-Index) as the ratio of the geometric means between post-implementation and baseline surveys. Binary prevalence variables were analysed by proportion estimation adjusted for robust SEs with clusters as villages. Change over time was analysed as the risk ratio between post-implementation and baseline. Statistical analysis was performed using Stata vs. 10 (StataCorp, 2005, College Station, Texas).

### Ethics

Extensive consultation was undertaken between the project team and community leaders, as well as liaison with village, district and provincial health staff. Village health workers provided participants with information regarding the surveys and signed informed consent was documented at the time of enrolment for the surveys. The survey team assisted the village health workers where participants expressed concerns or uncertainty relating to any aspect of their participation. The project was approved by the Human Research Ethics Committee of the National Institute of Malariology, Parasitology and Entomology (Hanoi, Vietnam), the Walter and Eliza Hall Institute of Medical Research (Melbourne, Australia) and Melbourne Health (Melbourne, Australia).

## Results

**Figure 1:**
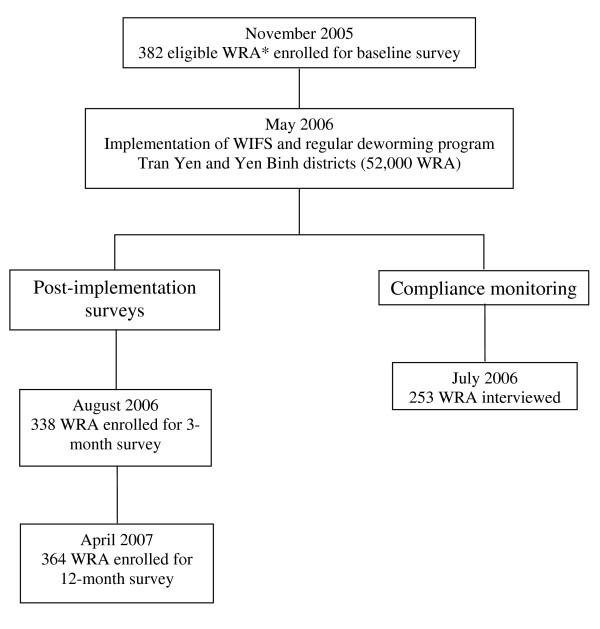
**Flow diagram of evaluation of WIFS ^# ^and 4 monthly deworming program: baseline, 3- and 12-months post-implementation**. ^# ^WIFS = Weekly Iron-Folic acid Supplementation * WRA = Women of Reproductive Age defined as aged between 15 – 45 years.

**Figure 2:**
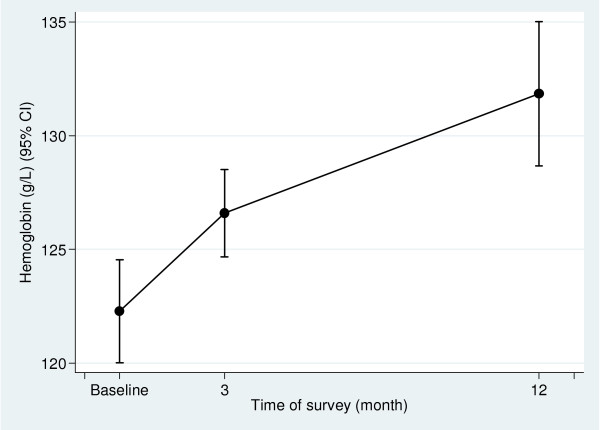
**Mean hemoglobin (95% CI) at baseline, 3- and 12-months post-implementation**.

**Figure 3:**
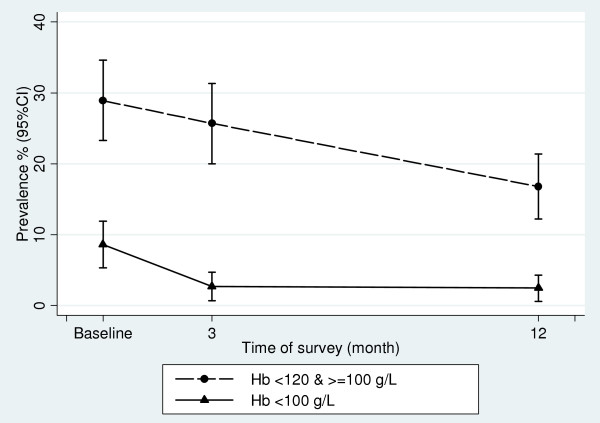
Prevalence of mild (Hb* <120 g/L & > = 100 g/L) and moderate/severe anemia (Hb <100 g/L), with 95% CI at each survey. * Hb = hemoglobin.

Three hundred and eighty-two women participated in the baseline survey, and 338 and 364 women attended the 3-month and 12-month post-implementation surveys respectively. Details of the study design and number of women enrolled in each survey are shown in Figure 1, and baseline demographic data are shown in Table 1. Altogether, 349/382 women had Hb measured at baseline, 335/338 in the three month and 363/364 in the 12 month survey. Figure 2 shows the Hb means and 95% confidence intervals at each survey point. The prevalence of anemia at baseline was 37.5% (131/349), (95% CI 31.3, 44.8), at three months post-intervention 28.4% (94/335) (24% reduction, RR 0.76, 95% CI 0.58, 0.98, p = 0.036) and 19.3% (70/363) at 12 months post-implementation, a reduction on baseline prevalence of 49% (RR 0.51, 95% CI 0.37, 0.71, p < 0.001). The prevalence of mild anemia (Hb* <120 g/L & > = 100 g/L) and moderate/severe anemia, (Hb<100 g/L) at baseline, three and 12 months post-implementation is shown in Figure 3.

**Table 1:** Demographic characteristics of women participating in the baseline survey in November 2005.

Demographic	Number of respondents	Mean ± SD or frequency (%)
Age (years)	382	30.1 ± 7.9
Number of children	378	1.7 ± 1.1
Education finished	375	
None/Primary (Grades 1–5)		84 (22.4)
Secondary (Grades 6–9)		211 (56.3)
High (Grades 10–12)/Post-secondary		80 (21.3)
Meat consumption (meals/week)	369	4.0 ± 2.9
Outdoor work (vs. indoor work)	372	317 (85.2)
Time since previous deworming	352	
1 wk-12 months		50 (14.2)
1 year to <10 years		160 (45.5)
≥ 10 years/never		142 (40.3)

**Figure 4:**
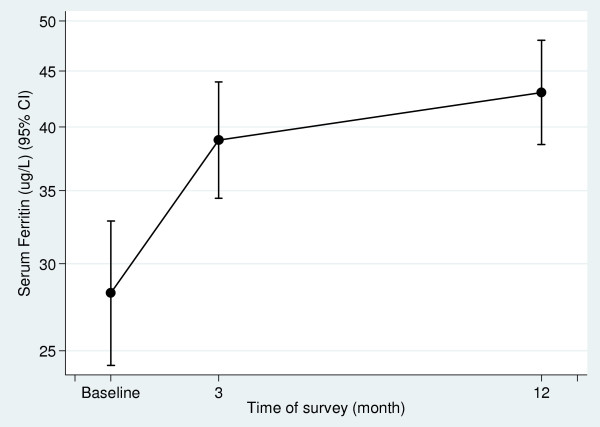
**Geometric mean serum ferritin (95% CI) at baseline, 3- and 12-months post-implementation**.

Geometric means for serum ferritin, soluble transferrin receptor and the TfR/F-Index are shown in Table 2. The serum ferritin geometric means and 95% CI are shown in Figure 4, mapped to a log scale. The prevalence of iron deficiency anemia at baseline was 14.0% (46/329) (95% CI, 10.2, 17.7). Three months post-implementation it had reduced to 5.9% (19/320) a reduction of 57% (RR 0.43, 95% CI 0.26, 0.70, p = 0.001) and at twelve months post-intervention was 4.5% (16/353) or 78% less than baseline (RR 0.32, 95% CI 0.15, 0.68, p = 0.003). Prevalence of iron deficiency with or without anemia was 22.8% (75/329) (95% CI 16.9, 28.6). By three months post-implementation this had reduced to 10.9% (35/320), a 52% reduction in prevalence (RR 0.48, 95% CI 0.34, 0.69, p < 0.001) followed by a further small reduction to 59% of baseline (RR 0.41, 0.26, 0.64, p < 0.001), a prevalence of 9.3% (33/353).

**Table 2:** Comparisons of iron status markers at baseline and 3- and 12-months post-implementation.

Survey	Serum ferritin μg/L*			Soluble Transferrin Receptor mg/L*			TfR/Fe-Index*		
	n	Mean	95% CI	n	Mean	95% CI	n	Mean	95% CI
Baseline	329	28.2	(24.2, 32.9)	336	1.69	(1.60, 1.78)	326	1.24	(1.05, 1.14)
3 month	320	38.9	(34.4, 44.0)	329	1.71	(1.63, 1.80)	320	1.10	(1.01, 1.07)
12 month	353	43.2	(38.6, 48.0)	361	1.41	(1.34, 1.48)	352	0.95	(0.92, 0.98)

* Exponentiated means and 95%CIs of log transformed values, SE adjusted for clusters as village.

The prevalence of hookworm infection by severity at baseline and at the 3- and 12-month post-implementation surveys is shown in Table 3. Overall prevalence of hookworm infection at baseline was 76.2% (279/366). At the three month post-implementation survey overall prevalence was 56.3% (126/224), a 26% reduction (RR 0.74, 95% CI 0.62, 0.89, p < 0.001) and at 12 months 23.0% representing a 70.0% reduction in prevalence (RR 0.30, 95% CI 0.24, 0.39, p < 0.001). The prevalence of any STH infection was 83.5% (95% CI 77.6, 89.5) at baseline, 64.3% (95% CI 55.7, 72.8) at three months and 32.7 (95% CI 25.0, 38.4) at twelve months post-implementation. Risk ratios for the change in prevalence of any soil transmitted infection at three- and twelve-months post-implementation were 0.77 (95% CI, 0.66, 0.89, p < 0.001) and 0.38 (95% CI, 0.30, 0.47, p < 0.001) respectively.

**Table 3:** Comparisons of prevalence of hookworm infection severity at baseline and 3- and 12-months post-implementation.

		Hookworm					
Survey		No infection*		Mild infection* (<2000 epg*)		Moderate/severe* infection (> = 2000 epg)	
	n	Percent	95% CI	Percent	95% CI	Percent	95% CI
Baseline	366	23.8	16.4, 31.1	60.1	54.1, 66.1	16.1	11.3, 20.9
3-month	224	43.2	34.8, 52.7	54.5	45.5, 63.4	1.8	0.04, 3.5
12-month	295	77.0	71.5, 82.5	21.6	16.2, 26.9	1.4	0.1, 2.6

epg = eggs per gram

* Proportion estimation by survey, SE adjusted for clusters as village

To assess compliance, the independent monitor interviewed 253 women of whom 251 had received at least some iron tablets. On a ten week recall, and supported by counting remaining tablets in the women's homes, two women reported not taking any iron tablets and 202/249 (71.1%) reported taking 6 or more tablets. High compliance was associated with literacy and education levels, 73% (154/211) of literate women reported high compliance compared with 50% (15/30) of illiterate women. For the deworming compliance, 225/241 (93.4%) of non-pregnant women received the albendazole and 217/225 (96.4%) of women receiving tablets reported taking it.

## Discussion

We have successfully integrated a large-scale, universal WIFS and deworming program into an existing public health structure. The program was distributed free of charge by village health workers to all women of reproductive age in the study districts. The post-implementation surveys showed an ongoing reduction in the prevalence and severity of anemia and hookworm infection over a twelve-month period. The independent monitor's report on the effectiveness of the distribution system and women's compliance supports the premise that the WIFS and regular deworming program was the principle reason for these improvements.

The strength of our approach was that distribution was easily integrated into existing health services and was made freely available to all women, regardless of socioeconomic or marital status. This was accompanied by nutritional education for communities and their health workers. This meant that the benefits of iron sufficiency were available for all women in their childbearing years, and their enthusiasm for the program was shown by the continued improvement in iron status and worm infection prevalence over twelve months, suggesting high compliance. The program was viewed as practical and effective by all levels of the health sector and the community and has now been expanded to every district in Yen Bai province.

The main limitation to the study was the absence of a control group. This would have allowed us to establish the prevalence of anemia and iron deficiency based on the proportion of women who responded to iron supplementation/deworming compared with those receiving placebo, and to be able to attribute response to the intervention. However, the main objective of the study was to conduct a large demonstration project using WIFS/deworming as an established strategy for reducing anemia and iron deficiency amongst women in developing countries, and we did not feel it ethical to include a control arm in which women were not treated for anemia and STH infections.

The residual anemia prevalence of 19.5% may be mainly explained by causes of anemia other than iron deficiency, such as genetic hemoglobinopathies, which are found amongst certain populations in South East Asia [25,26]. It has been estimated that in the WHO Western Pacific Region less than four percent of the population carry a Hb disorder [27] while a later study of schoolchildren in a different province of northern Vietnam found the prevalence of hemoglobinopathy (reporting totaled figures for HbA2, HbAE and HbF, others not tested for) to range between 3.8 and 9.3% [28]. Malaria is unlikely to have been a cause of anemia in the target population as it has been largely eliminated from the province, with only two imported cases reported in 2005 (Dr. L. B. Phu, Director Malaria Control Program, Yen Bai, Personal Communication). Compliance monitoring suggested that noncompliance was uncommon in the study group. However, to maintain high compliance we developed new training, educational and promotional materials for health workers and women following the 3-month compliance survey, as it showed that low and non-compliance with taking the weekly iron/folic acid supplement was associated with low education levels.

There have been few WIFS/multi-micronutrient programs successfully maintained for 12 months or more and most have targeted pregnant women [29,30] and/or adolescent schoolgirls and small children [29,31]. A WHO-sponsored program in three South-east Asian countries provided free daily iron supplements for pregnant women and encouraged non-pregnant women to purchase weekly iron supplements [15]. Extensive social marketing was undertaken within the targeted communities. In Vietnam, mean Hb levels increased by 6.0 g/L in a cohort of non-pregnant women tested four times over twelve months [32]. In Cambodia increases in Hb levels were associated with higher socio-economic status in groups of schoolgirls, garment factory workers and rural women [33], and in the Philippines there was a significant increase in serum ferritin but minimal change in Hb [34]. Dwivedi et al (2006) reported a Government-sponsored community-based WIFS program targeted at adolescent girls in 13 Indian states. A cross-sectional survey after 12 months showed a reduction in anemia prevalence ranging from 5% to 50% and, in one of the Indian states, a 70% reduction after two years of supplementation.

## Conclusion

In conclusion, our results show that free, universal WIFS with regular deworming was associated with a reduced prevalence and severity of anemia and hookworm infection in non-pregnant women over a 12-month period. The equitable, universal nature of the program and the endorsement and involvement of the health sector from provincial to village level were major factors in its ongoing success. Countries with high rates of anemia in women should urgently consider WIFS and regular deworming as interim measures until sustainable alternatives are implemented and shown to be effective [35,36].

## Competing interests

The authors declare that they have no competing interests.

## Authors' contributions

GC, TP, AM and BAB developed the project protocol, GC and BAB provided project management, GC, TP, and NT coordinated the project, TT coordinated the RTCCD surveys, GC and TP were responsible for managing the study teams, collecting data and data entry. GC, SM and LM assisted with data analysis. All authors reviewed and approved the final manuscript.

## Pre-publication history

The pre-publication history for this paper can be accessed here:


